# Inhibition of post-surgery tumour recurrence via a sprayable chemo-immunotherapy gel releasing PD-L1 antibody and platelet-derived small EVs

**DOI:** 10.1186/s12951-022-01270-7

**Published:** 2022-02-02

**Authors:** Jian Zhao, Hao Ye, Qi Lu, Kaiyuan Wang, Xiaofeng Chen, Jiaxuan Song, Helin Wang, Yutong Lu, Maosheng Cheng, Zhonggui He, Yinglei Zhai, Haotian Zhang, Jin Sun

**Affiliations:** 1 College of Pharmacy, Shenyang Pharmaceutical University, 103 Wenhua Road, Shenyang, 110016 Liaoning People’s Republic of China; 2 Department of Pharmaceutics, Wuya College of Innovation, Shenyang Pharmaceutical University, 103 Wenhua Road, Shenyang, 110016 Liaoning People’s Republic of China; 3 Multi-Scale Robotics Lab (MSRL), Institute of Robotics & Intelligent Systems (IRIS), ETH Zurich, 8092 Zurich, Switzerland; 4 Key Laboratory of Structure-Based Drug Design & Discovery of Ministry of Education, Shenyang Pharmaceutical University, Shenyang, 110016 China; 5 Department of Biomedical Engineering, School of Medical Devices, Shenyang Pharmaceutical University, Shenyang, 110016 Liaoning China; 6 School of Life Science and Biopharmaceutics, Shenyang Pharmaceutical University, 103 Wenhua Road, Shenyang, 110016 Liaoning People’s Republic of China

**Keywords:** Recurrence, Metastasis, Drug reservoir, Platelet-derived small EVs, PD-L1

## Abstract

**Background:**

Melanoma is the most serious type of skin cancer, and surgery is an effective method to treat melanoma. Unfortunately, local residual micro-infiltrated tumour cells and systemic circulating tumour cells (CTCs) are significant causes of treatment failure, leading to tumour recurrence and metastasis.

**Methods:**

Small EVs were isolated from platelets by differential centrifugation, and doxorubicin-loaded small EVs (PexD) was prepared by mixing small EVs with doxorubicin (DOX). PexD and an anti-PD-L1 monoclonal antibody (aPD-L1) were co-encapsulated in fibrin gel. The synergistic antitumour efficacy of the gel containing PexD and aPD-L1 was assessed both in vitro and in vivo.

**Results:**

Herein, we developed an in situ-formed bioresponsive gel combined with chemoimmunotherapeutic agents as a drug reservoir that could effectively inhibit both local tumour recurrence and tumour metastasis. In comparison with a DOX solution, PexD could better bind to tumour cells, induce more tumour immunogenic cell death (ICD) and promote a stronger antitumour immune response. PexD could enter the blood circulation through damaged blood vessels to track and eliminate CTCs. The concurrent release of aPD-L1 at the tumour site could impair the PD-1/PD-L1 pathway and restore the tumour-killing effect of cytotoxic T cells. This chemoimmunotherapeutic strategy triggered relatively strong T cell immune responses, significantly improving the tumour immune microenvironment.

**Conclusion:**

Our findings indicated that the immunotherapeutic fibrin gel could “awaken” the host innate immune system to inhibit both local tumour recurrence post-surgery and metastatic potential, thus, it could serve as a promising approach to prevent tumour recurrence.

**Graphical Abstract:**

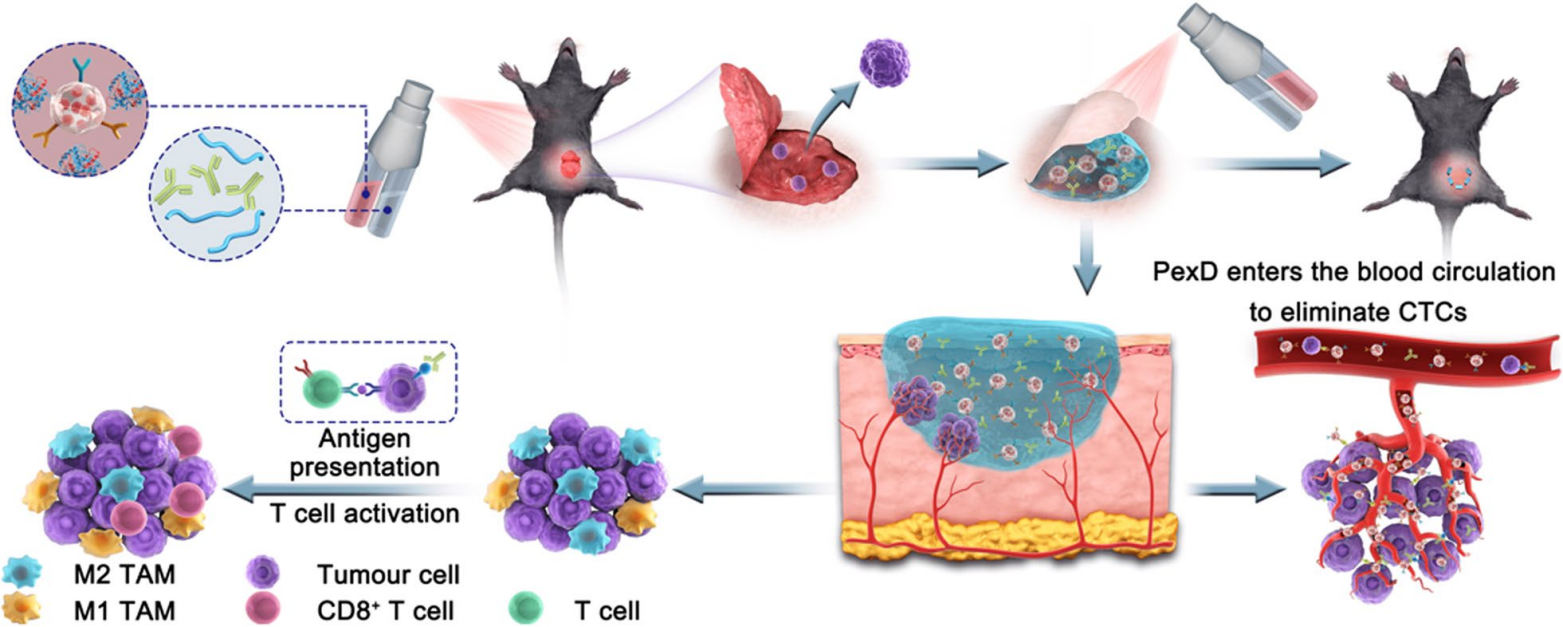

**Supplementary Information:**

The online version contains supplementary material available at 10.1186/s12951-022-01270-7.

## Background

Surgery is an effective method to treat melanoma, but unfortunately, local residual tumour micro-infiltration and systemic CTCs continue to cause tumour recurrence, resulting in patient death [1–4]. Immune checkpoint inhibitors (ICIs), especially PD-L1 blockers, have improved the efficacy of melanoma treatment and produced a lasting clinical response in some patients. However, systemic administration of ICIs promotes sustained clinical responses in less than 20% of patients with immunogenic tumours. The clinical efficacy of ICI monotherapy (such as aPD-L1 treatment) is limited due to a lack of immunogenic antigens and various immune resistance mechanisms [5–11].

Chemoimmunotherapy has been developed into one of the most effective combination therapeutic strategies for the treatment of malignant cancer [12, 13]. Chemotherapeutic drugs (such as DOX) can directly kill tumour cells and induce ICD to generate tumour antigens or danger signals; subsequently, the antitumour immune response can be induced by co-stimulation with tumour antigens and an ICI [14, 15]. However, safe and effective targeted delivery of chemotherapeutic drugs remains challenging, in part because of poor bioavailability and non-specific targeting. Thus, the ability to combine safe and effective delivery of chemotherapeutic drugs with immune checkpoint blockade is critical to prevent tumour recurrence and metastasis after surgery [8, 16].

Small EVs are naturally occurring extracellular vesicles with a size range of 40 to 160 nm (average ~ 100 nm) in diameter, making them much smaller than cells [17–20]. Inspired by the natural targeting of platelets to CTCs, we speculated that platelet-derived small EVs (Pex) have the same superior CTCs capture ability as platelets. We loaded DOX in Pex with the aim of neutralizing tumour cells through the specific adhesion interaction between Pex and CTCs, thereby inhibiting tumour metastasis. Pex are also smaller than platelets and can deliver drugs more effectively [15, 21, 22].

**Fig. 1:**
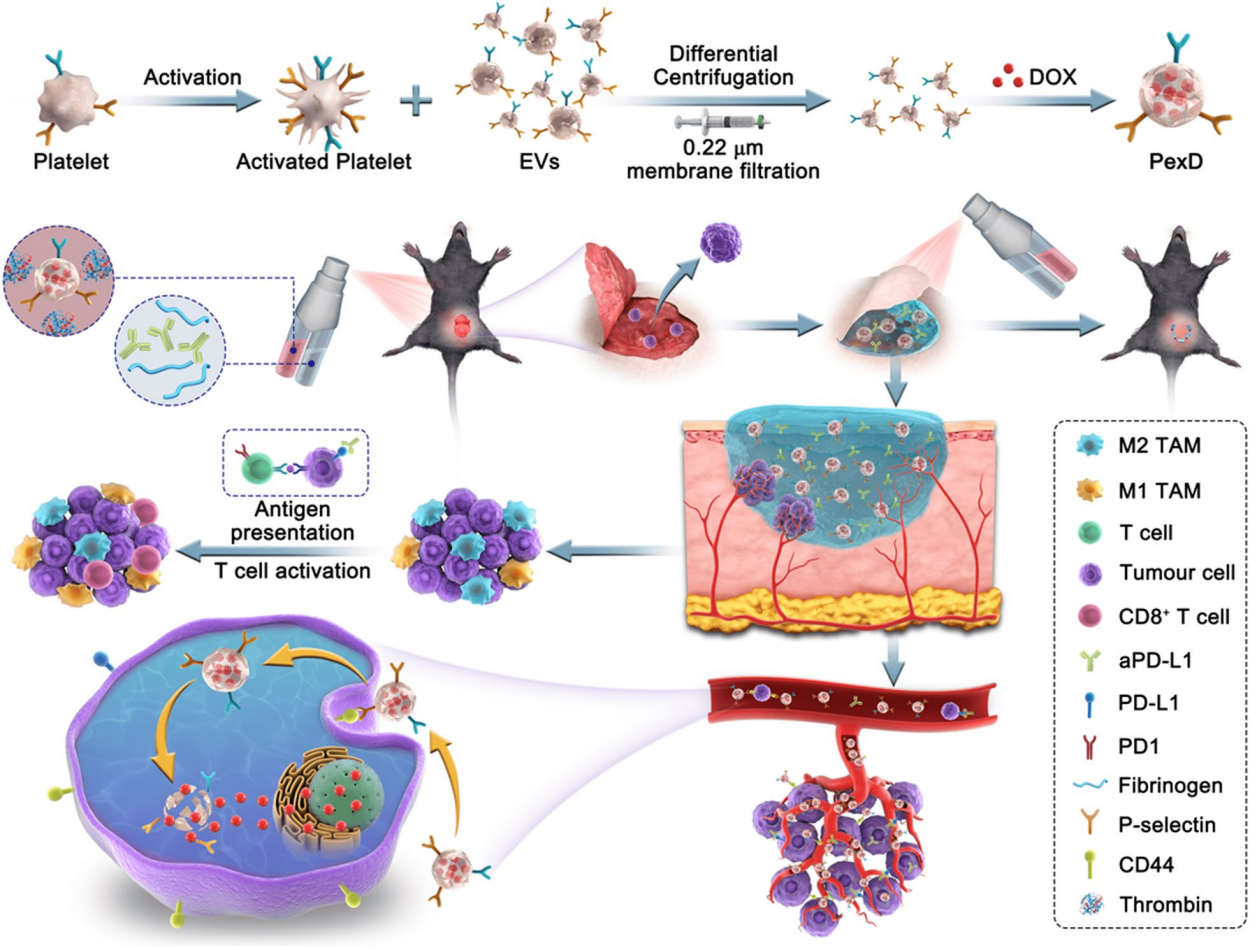
The schematic diagram showed a bioreactive fibrin gel containing PexD biomimetic nanoparticles and aPD-L1 sprayed in situ in the tumour bed after surgery. Combining chemotherapy and immunotherapy to eliminate residual in situ tumour cells and capture circulating tumour cells prevents melanoma recurrence and metastasis

Here, we developed a combination of chemotherapy and immunotherapy based on the use of an in situ-formed bioreactive gel as a drug reservoir. Fibrin gel is a pharmaceutical material approved by the FDA and is formed by the interaction between fibrinogen and thrombin [23, 24]. The utilized post-operative spraying method and the creation of a temporary shield enabled connection with and protection of injured tissue to promote wound healing [25–28]. In view of this, PexD was added to a thrombin solution, and aPD-L1 was added to a fibrinogen solution, which could be sprayed by using a dual-cartridge sprayer. The sprayable gel acted as a reservoir to concentrate and gradually release PexD and aPD-L1 after surgical resection of the tumour. PexD could induce tumour ICD and promote antitumour immune responses, while also entering the blood circulation through damaged blood vessels in situ to track and adhere to CTCs. aPD-L1 was also released to block the PD1/PD-L1 pathway (Fig. 1). The combination of both strategies triggered relatively strong T cell immune responses. Overall, the developed gel created a favourable environment in which PexD eliminated residual tumour cells in situ and CTCs to prevent tumour recurrence [29–35].

## Results and discussion

### Characterization of Pex and PexD

**Fig. 2:**
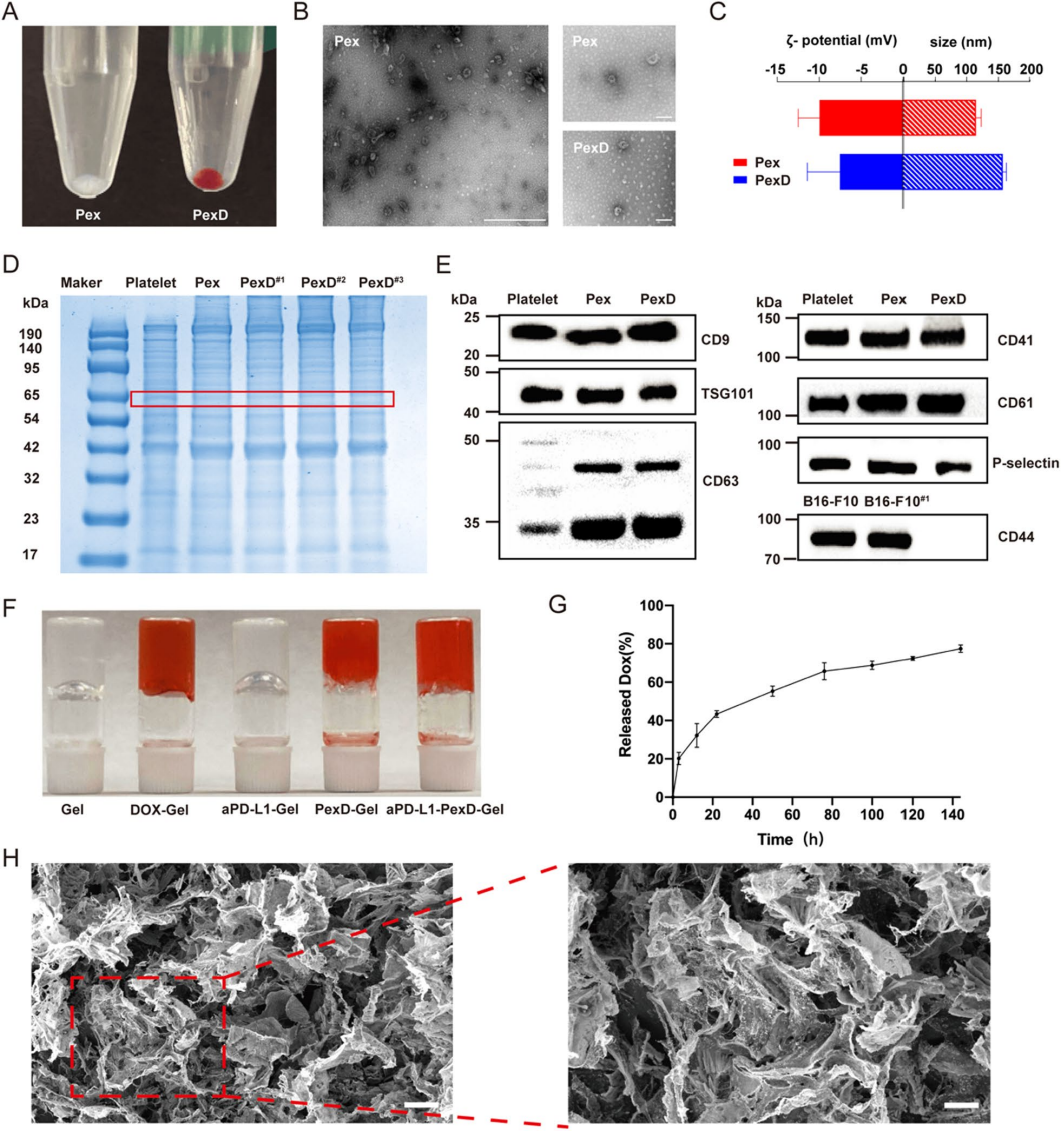
Characteristics of PexD and gel. **A** Photos of Pex and PexD. **B** TEM images of Pex and PexD. Scale bar (left side): 500 nm. Scale bar (right): 100 nm. **C** Particle size and potential of Pex and PexD. **D** SDS-PAGE protein analysis of Platelets, Pex, and PexD. **E** Western blot analyses were performed on Platelet, Pex, PexD labeled CD41, P-selectin and CD61, CD9 CD63 and TSG101 characteristic of Pex and PexD, and CD44 on B16-F10 cells. **F** Photos of Platelet, Pex and PexD. **G** DOX curve of aPD-L1-PexD-Gel in PBS at pH 6.5. Data are expressed as mean ± SD (n = 3). **H** Cryo-scanning electron microscopy (SEM) images of fibrin gel containing PexD nanoparticles and aPD-L1. Scale bar (left side): 40 µm. Scale bar (right): 20 µm

We purified Pex by ultracentrifugation, and PexD was prepared by mixing the small EVs with DOX. The Pex obtained after ultra-high-speed centrifugation were a white precipitate, and PexD was red due to the loaded DOX (Fig. 2A). Morphological investigation of small EVs before and after DOX loading was performed by transmission electron microscopy (TEM) (Fig. 2B). Pex and PexD both showed characteristic saucer-like bilayer membrane structures, demonstrating that Pex remained intact after loading with DOX. A Malvern particle size analyser (Fig. 2C) indicated that the small EVs had a relatively narrow size distribution, and the mean diameter was approximately 115 nm for free small EVs. After loading with DOX, the mean particle size increased to 157 nm. The average zeta potential of PexD was higher than that of Pex due to the positively charged DOX (Fig. 2C).

Analysis of the membrane protein marker sequence by SDS-PAGE indicated that the exclusive proteins inherited from Pex and platelets were well preserved in the PexD protein profile (Fig. 2D). The proteins on the surface of Pex were not affected by DOX loading. Next, Western blotting was used to detect the essential protein expression of platelets, Pex and PexD (Fig. 2E). Similar to purified small EVs obtained by differential ultracentrifugation, PexD also showed expression of the exosomal biomarkers TSG101, CD9 and CD63. P-selectin is a cell-adhesion molecule that binds to CD41 and CD61 to produce the key platelet adhesion molecule integrin αIIbβ3 [36]. These proteins coexisted on platelets, Pex and PexD. The CD44 protein that binds to P-selectin was also found to be significantly expressed on B16-F10 cells.

### In situ formation of fibrin gel by spraying

After spraying fibrin gel containing PexD and aPD-L1, a red hydrogel was formed with the overlays exhibiting the characteristic colours of orange PexD and transparent aPD-L1 (Fig. 2F). The morphology of the fibrin gel containing PexD nanoparticles and aPD-L1 was validated by a rheology test. In the dynamic time sweep (Additional file 1: Fig. S1A), the value of the storage modulus (Gʹ) was consistently greater than that of the loss modulus (Gʺ), indicating that the hydrogel was a steady soft material. In the dynamic strain sweep (Additional file 1: Fig S1B), the values of Gʹ dominated those of Gʺ, and the critical strain value of the gel was 68.35%, indicating robust gel formation. In the dynamic frequency sweep (Additional file 1: Fig S1C), the gel behaved independently of the frequency in the region of 0.1–100 rad s^−1^. Excellent mechanical properties guaranteed gel stability in vivo. Gel morphology was characterized with scanning electron microscopy (Fig. 2H). The gel exhibited a three-dimensional porous structure, which was a prerequisite for drug release.

We further explored the DOX-release profile of gels. We added PexD to the gel, incubated the drug-loaded gel in the tumour microenvironment (PBS at pH 6.5) and then quantified DOX release from the gel at different time points. From the release curve (Fig. 2G), a cumulative release of 43.4% of the DOX in the gel was observed within 24 h. The results showed that the release of DOX from the drug-loaded gel system was a time-dependent delivery process. Finally, based on the observation of mouse kidney adhesion to the gel, the gel had high adhesion and the potential to adhere to the tumour resection site (Additional file 1: Fig S2).

### In vitro adhesiveness, cellular uptake, cytotoxicity, and ICD induction of PexD

**Fig. 3:**
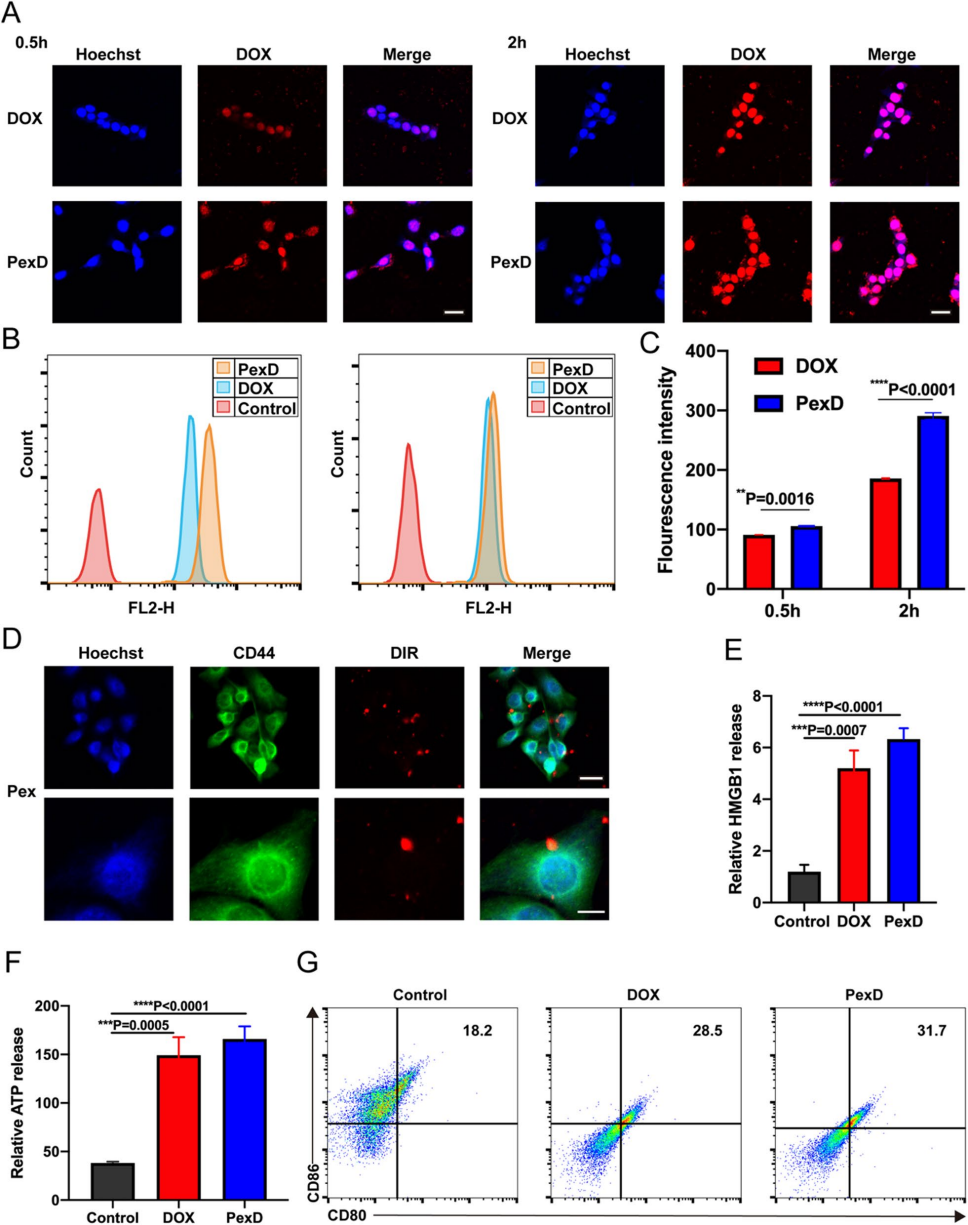
In vitro adhesiveness, cellular uptake, cytotoxicity, and ICD of evaluation of PexD. **A** Confocal microscope images of B16-F10 cells incubated with free DOX and PexD for 0.5 and 2 h, respectively. Scale bar: 10 μm. **B** Flow cytometry measurement of B16-F10 cells incubated with free DOX and PexD for 0.5 and 2 h. Scale bar: 10 µm. **C** Fluorescence intensity analyzed by flow cytometry. The data are expressed as mean ± SD (n = 3). **D** The adhesion of DiR-labeled Pex to B16-F10 cells with confocal laser scanning microscope. CD44 staining (green), DIR-labeled Pex (red) and nucleus (blue). Scale bar (upside): 10 µm. Scale bar (down): 5 µm. **E** Relative HMGB1 release from B16-F10 cells treated for 24 h with PexD or DOX (3 μg mL^−1^). The data are expressed as mean ± SD (n = 3). **F** Relative ATP release from B16-F10 cells treated for 24 h with PexD or DOX (3 μg mL^−1^). The data are expressed as mean ± SD (n = 3). **G** Representative flow cytometric analysis of CD80^+^ CD86^+^ cells. The data are expressed as mean ± SD (n = 3). **C**–**F** **P < 0.01; ***P < 0.001; ****P < 0.0001

Due to the strong affinity between P-selectin and the receptor CD44, we explored the adhesion of Pex to B16-F10 cells. We explored the adhesion between DiR-labelled Pex and B16-F10 cells through confocal laser scanning microscopy, and observed co-localization of DiR-labeled Pex with CD44 in B16F10 cells (Fig. 3D). The results indicated that Pex could adhere to the surface of tumour cells. This finding further confirmed that the receptor CD44 was involved in the recognition process between Pex and tumour cells. It has been reported that a specific and strong affinity exists between the receptor CD44 and P-selectin [6]. Therefore, we determined the expression levels of the key protein P-selectin on PexD and found that PexD maintained high expression of P-selectin, similar to platelets. The high affinity between P-selectin on the surface of Pex and surface-expressed CD44 on tumour cells would help PexD recognize and capture tumour cells and CTCs.

To study whether biomimetic PexD nanoparticles can increase the cellular internalization of DOX, we incubated B16-F10 cells with free DOX and PexD for 0.5 h and 2 h (Fig. 3A). The fluorescence intensity of DOX at 2 h in each group was significantly greater than that at 0.5 h, proving that the uptake of DOX by tumour cells increased in a time-dependent manner. The cellular uptake efficiency of PexD was higher than that of free DOX at the same incubation time, which could be attributed to the ready binding of Pex with B16-F10 cells. We further used flow cytometry to quantitatively determine the cellular uptake of DOX from PexD and a free DOX solution by B16-F10 cells. As shown in Fig. 3B, C, PexD showed enhanced B16-F10 cellular uptake of DOX compared to the free DOX solution. These results were consistent with the results of fluorescence microscopy observations.

The in vitro cytotoxicity of PexD and the free DOX solution to B16-F10 cells was determined by using the 3-(4,5-dimethylthiazol-2-yl)-2,5-di-phenyl tetrazolium bromide (MTT) assay. When compared to the free DOX solution, PexD had significantly higher cytotoxicity (Additional file 1: Fig S3). The half-maximal inhibitory concentration (IC_50_) values of the free DOX solution and PexD were calculated to be 0.341 and 0.115 µg mL^−1^, respectively. Collectively, based on the higher cellular uptake of PexD, the results showed that PexD had high cytotoxicity to B16-F10 cells.

DOX is a well-known ICD-inducing chemotherapeutic drug that can increase the cell-surface exposure of calreticulin and extracellular release of HMGB1 and ATP. Accordingly, we examined the ability of PexD to induce ICD in B16-F10 cancer cells in vitro by assessing the cell-surface expression of calreticulin and extracellular secretion of HMGB1 and ATP. Importantly, at 24 h post incubation, B16-F10 cells treated with 3 µg mL^−1^ free DOX induced lower levels of calreticulin expression than PexD, showing that PexD could induce stronger ICD in cancer cells (Additional file 1: Fig S4). PexD induced higher calreticulin expression in B16-F10 cells than the DOX solution. Additionally, the amount of released HMGB1 in the cell culture medium of B16-F10 cells at 24 h post incubation was higher after treatment with PexD than after treatment with free DOX (Fig. 3E). The amount of ATP released into the cell culture medium from B16-F10 cells treated with PexD for 24 h was also higher than that released from free DOX-treated B16-F10 cells (Fig. 3F). These results indicated that, compared with free DOX, PexD induced stronger ICD in B16-F10 cancer cells, agreeing well with the abovementioned cellular uptake and cytotoxicity results.

Then we examined ICD-induced maturation of dendritic cells (DCs). To assess the maturation status of DCs, bone marrow-derived immature DCs isolated from B16F10 tumour-bearing C57BL/6 mice were co-cultured with B16F10 cancer cells that were pretreated with PexD or DOX. Importantly, the frequency of matured DCs (CD11c^+^/CD80^+^/CD86^+^) significantly increased compared to DCs co-cultured with non-treated B16-F10 cancer cells, indicating that PexD efficiently promoted DC maturation via ICD of cancer cells (Fig. 3G, Additional file 1: Fig S5). These findings indicated that PexD elicited ICD in cancer cells and efficiently promoted DC maturation. The interaction of CD80 or CD86 with the T-cell receptor, CD28, promoted T-cell responses by enhancing the expansion of antigen-specific T cells. CD80 and CD86 had also been shown to regulate Th1/Th2 differentiation [37–39].

### In vivo eradication of CTCs in the circulatory system

CTCs in the blood are the main cause of tumour metastasis. To test the ability of PexD to capture CTCs, saline, DOX, platelet-DOX, and PexD (40 µg DOX per mouse) were administered intravenously to separate mice, and then B16-F10 cancer cells were injected into the C57BL/6 mice via the tail vein to simulate CTCs. After 12 days, the mice were sacrificed, and the lungs were isolated. As shown in Additional file 1: Fig S6, lung micro-metastasis was most common in the mice treated with saline. Compared with the mice treated platelet-DOX, those treated with PexD showed almost no metastatic pulmonary nodules, implying a better CTC capture efficiency. This result was attributed to the small particle size of PexD, which could better penetrate deeply into the tumour tissue. In the blood circulation, the high affinity between P-selectin and CD44 helped PexD capture CTCs and eliminate these cells through the subsequent release of DOX.

### In vivo biodistribution

To evaluate the capacity of Pex to accumulate at the residual tumour cells, B16-F10 tumour-bearing C57BL/6j mice were employed. The mice were intravenously injected immediately with DiR-labelled Pex or free DiR at a dose of 1 mg kg^−1^ equivalent to DiR after incomplete removal of tumours. As shown in Additional file 1: Fig. S7A, DiR-labelled Pex showed higher fluorescent signals within the tumours than did free DiR, suggesting the tumour-seeking ability of Pex, and the highest accumulation in tumours was at 3 h post-injection. Then the residual tumour and major organs were harvested at 3 h and 12 h post-injection, respectively. The mice treated with free DiR exhibited negligible fluorescent signals in residual tumours and high fluorescent signals in livers, spleens, and lungs (Additional file 1: Fig. S7B). The fluorescent intensities of tumours treated with Pex were 7.22- and 5.81-fold higher than those treated with free DiR at 3 h and 12 h, respectively (Additional file 1: Fig. S7C).

### Immunotherapy gel inhibits tumour recurrence

**Fig. 4:**
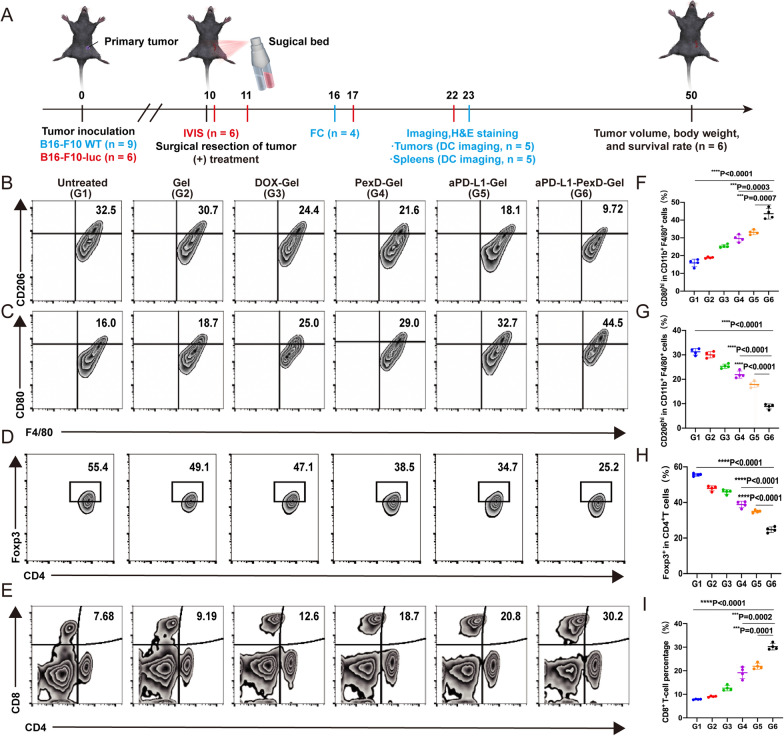
Six days after treatment, immune responses induced by aPD-L1-PexD-Gel B16-F10 tumours were harvested from mice. **A** Schematic diagram of treatment in a distal surgical tumour model. (B16-F10-WT: wild type B16-F10 melanoma cells; B16-F10-luc: luciferase-tagged B16-F10 melanoma cells; IVIS: in vivo imaging system; FC: flow cytometry analysis; DC imaging: digital camera imaging). **B** Representative flow cytometry analysis images of M2 macrophages (CD206hi) and **C** M1 macrophages (CD80hi) on gated F4/80^+^ CD11b^+^ CD45^+^ cells. **D** Representative flow cytometric analysis of CD4^+^ Foxp3^+^ T cells on CD3^+^ cells. **E** Representative flow cytometric analysis of CD8^+^ T cells on CD3^+^ cells. **F** Relative quantification in **B**. The data are expressed as the mean ± SD (n = 4). **G** Relative quantification in **C** is expressed as the mean ± SD (n = 4). **H** Relative quantification in **D** is expressed as the mean ± SD (n = 4). **I** Relative quantification in **E** is expressed as the mean ± SD (n = 4). **F**–**I** *P < 0.05; **P < 0.01; ***P < 0.001; ****P < 0.0001

To verify the therapeutic effect of aPD-L1-PexD-Gel, we used an incomplete tumour resection model. Different types of fibrin gel including gel, DOX-Gel, aPD-L1-Gel, PexD-Gel, and aPD-L1-PexD-Gel (40 µg DOX per mouse, 40 µg aPD-L1 per mouse) were sprayed into the tumour resection cavity in situ (Fig. 4A). As shown in Fig. 4D, E, H and I we observed a reduction in regulatory T cell (Treg cells: CD4^+^ Foxp3^+^ T cells, also known as suppressor T cells) levels and elevated tumour-infiltrating cytotoxic T lymphocyte (CD8^+^ T cells) levels in the groups treated with aPD-L1-Gel or PexD-Gel. These findings implied that both the PD-L1 blockade strategy using aPD-L1 treatment and the PexD strategy dependent on tumour destruction could trigger T cell-mediated immune responses.

Notably, the combination of both strategies triggered stronger T cell immune responses. The optimal result, i.e., T cells being revitalized to the greatest extent, was obtained when aPD-L1-PexD-Gel was used. Because the initial DOX release from the gel induced tumour ICD and then the dying tumour cells operated like a “tumour vaccine”, aPD-L1 could reactivate nonfunctional T cells by blocking the PD-1/PD-L1 signalling pathway.

We observed a significant decline (25.2%) in the proportion of M2-like macrophages and a significant increase (44.5%) in the proportion of M1-like macrophages in the aPD-L1-PexD-Gel-treated group compared with the other treatment groups and the gel control group (Fig. 4B, C, F, G) Phenotypic transformation of M2-like tumour-associated macrophages (TAMs) into M1-like TAMs could block the TAM-mediated formation of tumour lymphatic vessels and blood vessels to inhibit the processes of tumour metastasis and recurrence.

**Fig. 5:**
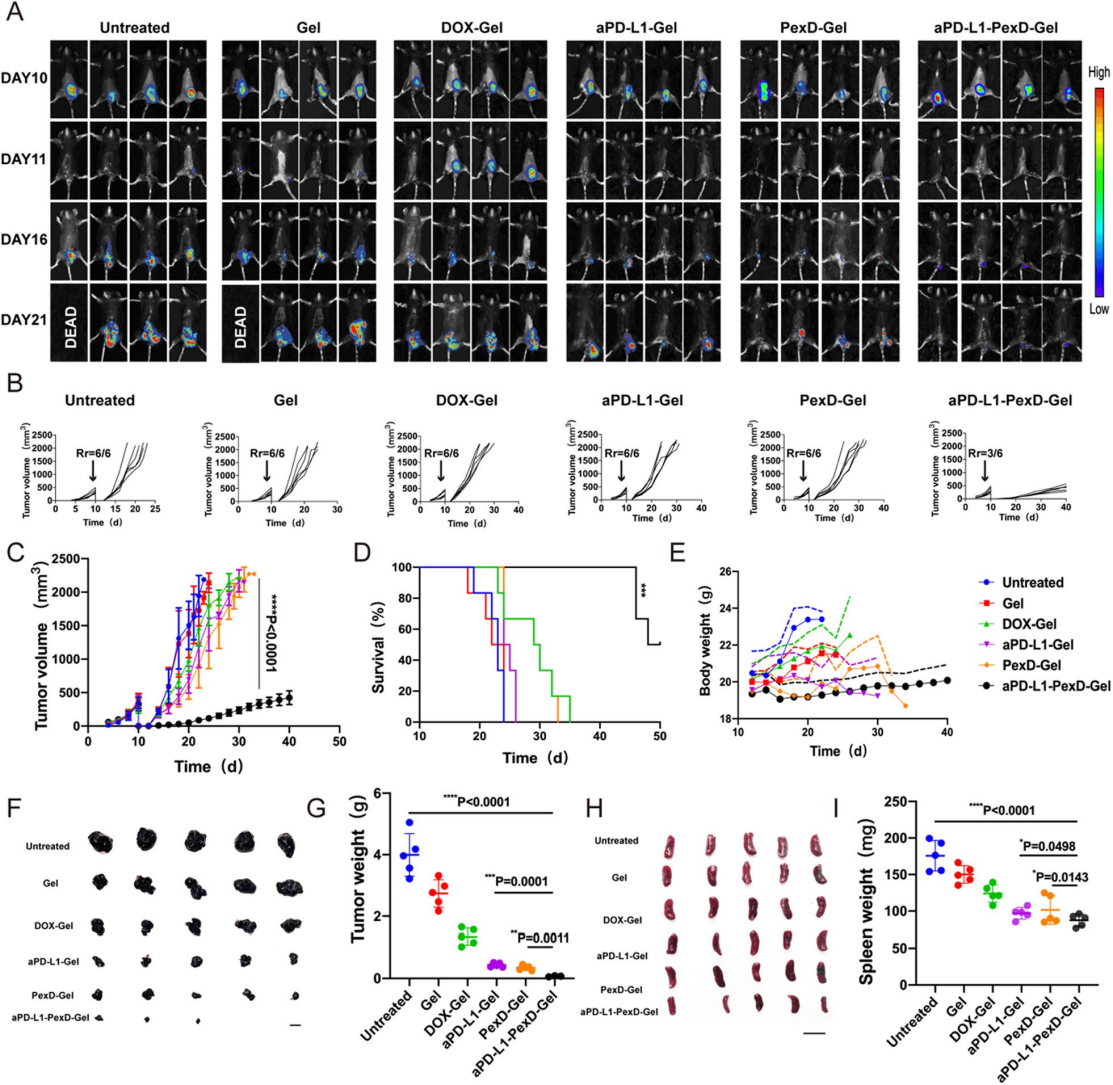
The gel could prevent B16-F10 tumour recurrence after surgery. **A** In vivo bioluminescence imaging of B16-F10 tumour after primary tumour removal. Each treatment group showed four representative mice. The images related to day 10 were taken before the operation. **B**, **C** Average tumour growth kinetics in different groups. When the first mouse in the respective group died, the growth curve stopped. The data are expressed as mean ± SD (n = 6). Statistical significance is obtained through multiple comparisons between one-variance analysis and Tukey’s post-hoc test. **D** After the various treatments, the survival rate corresponded to the tumour size of the mice. The data are expressed as mean ± SD (n = 6). **E** Changes in body weight of mice in different groups. The data are expressed as mean ± SD (n = 4). **F**, **H** Tumours and spleens after different treatments (n = 5). Scale bars: 1 cm. **G**, **I** Quantitative graph of tumour and spleen weight. The data are expressed as mean ± SD (n = 5). **G**, **I** *P < 0.05; **P < 0.01; ****P < 0.0001

Tumour growth was monitored by measuring the bioluminescence signal from B16-F10-luc cancer cells (Fig. 5A). Three out of six mice exhibited no detectable tumours after treatment with aPD-L1-PexD-Gel, implying relatively good tumour growth control (Fig. 5B, C). The images and weights of recurrent tumours (Fig. 5F, G) also indicated that aPD-L1-PexD-Gel showed advantages in local tumour recurrence prevention. Fifty percent of mice treated with aPD-L1-PexD-Gel survived for at least 50 days (Fig. 5D), and the body weight of these mice was not affected by the treatment (Fig. 5E). Furthermore, we found that the aPD-L1-PexD-Gel-treated group had the smallest tumours, and tumours, spleens, and lungs collected on day 22 showed that tumour metastases had been eliminated in this group (Additional file 1: Fig S8). These results were consistent with the in vivo bioluminescence imaging results shown in Fig. 5A.

Therefore, aPD-L1-PexD-Gel was concluded to represent a very effective immunogel drug that could prevent tumour recurrence and metastasis. In addition, compared with healthy mice, tumour-bearing mice had obvious differences in spleen weight, which were caused by abnormal immune function. Tumour-bearing mice usually showed compensatory splenomegaly. Therefore, we euthanized experimental mice and harvested the spleen to compare the degree of splenomegaly among various groups. aPD-L1-PexD-Gel had a good therapeutic effect, and the spleen size of mice given this treatment was close to that of healthy mice. The spleen size of mice in other groups showed a remarkable increase. Spleen weight was further quantified. The spleen weight of mice treated with saline was 1.7 times higher than that of mice treated with aPD-L1-PexD-Gel (Fig. 5H, I), further proving that aPD-L1-PexD-Gel produced good antitumour immunity.

### Immunotherapy gel for the treatment of distant tumours

**Fig. 6:**
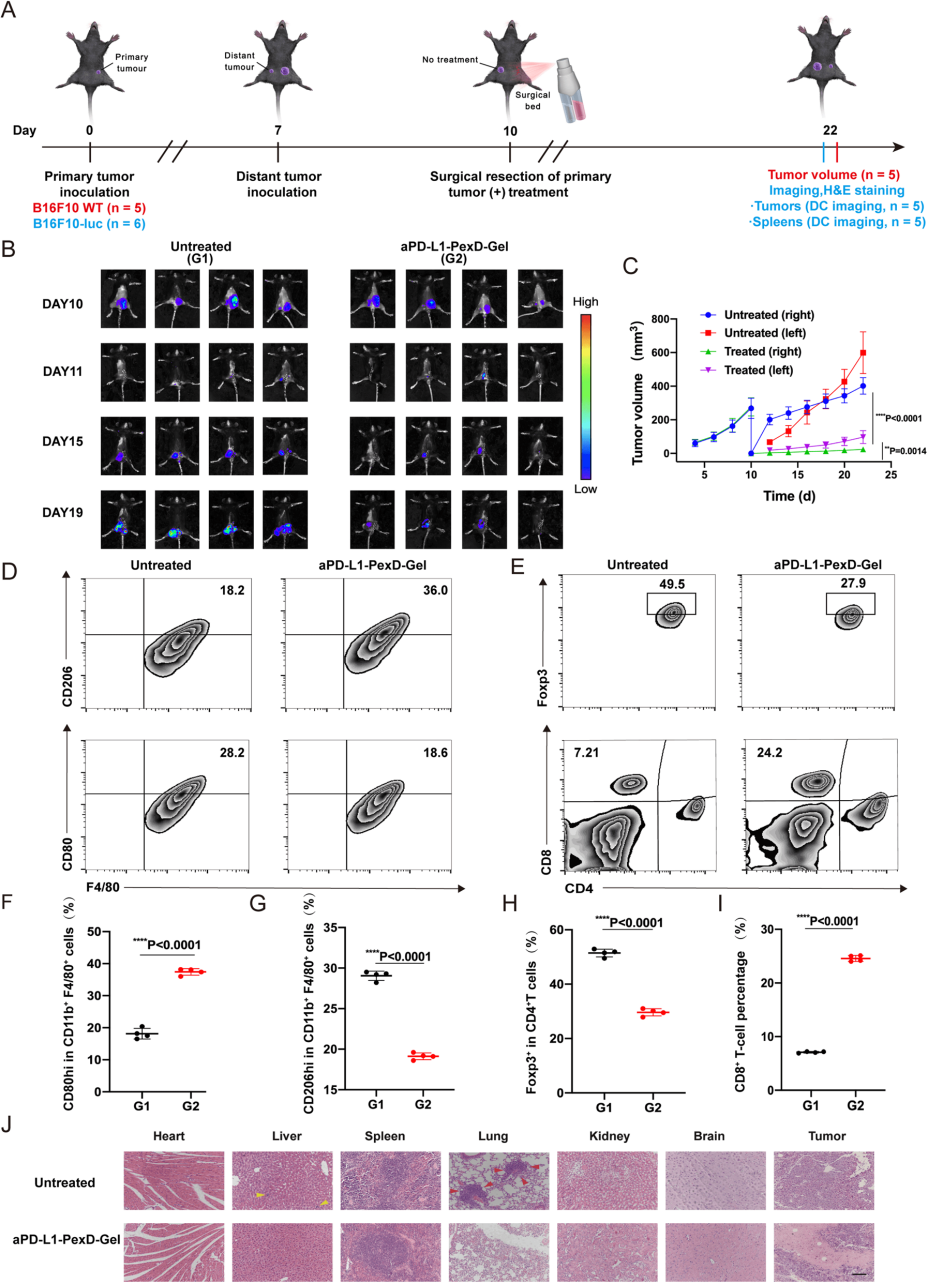
Local treatment of aPD-L1-PexD-Gel on systemic anti-tumour immune response. **A** Schematic diagram of treatment in a distal surgical tumour model. (B16-F10-WT: wild type B16-F10 melanoma cells; B16-F10-luc: luciferase-tagged B16-F10 melanoma cells; DC imaging: digital camera imaging). **B** B16-F10 tumour bioluminescence imaging in vivo in response to local aPD-L1-PexD-Gel treatment. **C** Growth curves for left and right tumours in untreated and treated mice. Data are presented as mean ± SD (n = 5). **D** Representative flow cytometry analysis images of M2 macrophages (CD206hi) and M1 macrophages (CD80hi) on gated F4/80^+^ CD11b^+^ CD45^+^ cells. **E** Representative flow cytometric analysis of CD4^+^ Foxp3^+^ T cells and CD3^+^ CD8^+^ T cells. **F**, **G** Relative quantification in **D**. The data are expressed as the mean ± SD (n = 4). **H**, **I** Relative quantification in **E**. The data are expressed as the mean ± SD (n = 4). **J** After various treatments, the entire lung India ink staining and H&E staining photos of tumour, kidney, lung, spleen, liver, and heart sections were collected. The yellow and red arrows represented metastases to the liver and lung, respectively. Scale bars: 1 mm. **F**–**I** ****P < 0.0001

To confirm that aPD-L1-PexD-Gel activated local innate immunity to inhibit tumour development, we investigated whether local treatment with aPD-L1-PexD-Gel triggered systemic immune responses to inhibit distant tumours. B16-F10 cancer cells were inoculated into the side opposite the primary tumour to model tumour metastasis. The primary tumour was partially excised, and fibrin gel containing PexD nanoparticles and aPD-L1 (40 μg DOX per mouse, 40 μg aPD-L1) was sprayed on the excision site (Fig. 6A). We observed that aPD-L1-PexD-Gel inhibited both local tumour recurrence and tumour growth at the distant site (Fig. 6B). The tumour growth curve (Fig. 6C) as well as the images and weights of recurrent tumours (Additional file 1: Fig S9A, C) indicated that aPD-L1-PexD-Gel could trigger systemic immune responses and produce obvious tumour recurrence inhibition.

For flow cytometric analysis, distant tumours and blood were collected and pooled to form single-cell suspensions for testing. Consistent with the above findings, the level of CD8^+^ T cells in the blood of mice sprayed with aPD-L1-PexD-Gel was increased significantly, while the level of Foxp3^+^ T cells was reduced remarkably (Fig. 6D, F, G). The numbers of M1-like TAMs were increased in distant tumours, while the numbers of M2-like TAMs were decreased (Fig. 6E, H, I). Compared with those in the saline group, the mice in the aPD-L1-PexD-Gel group had normal spleens (Additional file 1: Fig S9B, D), and haematoxylin and eosin (H&E) staining (Fig. 6J) showed that there was basically no tumour metastasis in the main organs after aPD-L1-PexD-Gel treatment. These findings were consistent with the results for the above tumour resection model, indicating that aPD-L1-PexD-Gel could inhibit tumour recurrence at the primary site and suppress tumours at the distant site, thereby further confirming the activation of the immune system.

## Conclusions

In summary, we developed a simple strategy for post-surgical cancer immunotherapy by spraying an in situ-formed therapeutic gel at the tumour resection site. The gel could facilitate the reversal of the immunosuppressive tumour microenvironment and induce systemic immunological responses to inhibit both local recurrence and systemic development. PexD nanoparticles embedded in a gel matrix could be released in a controlled manner, eliminating tumours in situ and entering the bloodstream to adhere to and kill CTCs. PexD could induce tumour ICD, and then the dying tumour cells could operate like a “tumour vaccine” to elicit a tumour-specific immune response to eliminate residual tumour cells. In addition, due to the role of aPD-L1 in inhibiting the PD-1/PD-L1 pathway and restoring the killing activity of cytotoxic T cells, the simultaneous release of aPD-L1 at the tumour site could significantly enhance tumour suppression. The combination of both strategies triggered even stronger T cell immune responses. In terms of chemoimmunotherapy, the tumour microenvironment was remodelled away from immunosuppression, including increased numbers of cytotoxic CD8^+^ T lymphocytes and M1-like TAMs and decreased numbers of Tregs and M2-like TAMs, all of which predicted favourable therapeutic responses. The proposed sprayable chemoimmunotherapy gel offers a promising way to improve clinical benefit by specifically killing cancer cells and potentiating checkpoint blockade immunotherapy.

## Materials and methods

### Preparation and characterization of PexD

Platelets were obtained by centrifuging fresh mouse blood at 200*g*, suspending the blood in the same volume of ACD solution (citric acid–glucose), and then centrifuging it at 800*g*. To prepare Pex, platelets were diluted with Tyrode-HEPES buffer (1 mM MgCl_2_, 2 mM CaCl_2_, and 3 mM KCl_2_) to 250 × 10^6^ platelets mL^−1^, combined with Ca^2+^ ionophore (10 mM, Sigma-Aldrich) at 30 °C, incubated for 30 min, and then centrifuged at 800*g* for 10 min. The collected supernatant was further ultracentrifuged, and the extracellular vesicles were ultracentrifuged at a speed of 100,000*g* for 90 min to concentrate the particles. After resuspension, the extracellular vesicles were passed through a 220-nm microporous membrane. The protein concentration of Pex was quantified, and the weight of the membrane was twice the weight of the membrane protein. PexD was prepared by mixing small EVs with DOX. DOX was properly diluted with sterile saline for injection. A mixture of 500 μL DOX (1 mg mL^−1^) and 500 μL small EVs solution (2 mg mL^−1^) was prepared at 37 °C and incubated for 1 h, and then the mixed solution was added to a centrifuge tube at 4 °C. PexD was obtained by centrifugation at a 100,000 rpm for 90 min. By detecting the absorbance value at 490 nm, we calculated the DOX load in small EVs by drawing a standard DOX curve. The morphology of Pex and PexD was then examined using TEM.

### Western blot analysis of key proteins of platelets, Pex, and PexD

A protein extraction kit (Ding Guo, China) was used to extract total cell protein. The extracted proteins from platelets, Pex and PexD were boiled for 5 min and separated by SDS-PAGE. Coomassie blue staining was used to visualize proteins. Proteins were prepared for western blotting as previously stated. Following gel electrophoresis, proteins were transferred to polyvinylidene fluoride membranes (Bio-Rad). After 2 h of blocking in 5% skim milk, the membranes were incubated with anti-TSG101 (Abcam, ab125011), anti-CD9 (Abcam, ab92726), anti-CD63 (Abcam, ab217345), anti-CD61 (Abcam, ab119992), anti-CD41 (Abcam, ab63983) or anti-P-selectin (Abcam, ab6632). The antibodies were added at a dilution of 1:1000 and incubated at 4 °C overnight, and the blots were then incubated with 5% skim milk for 2 h. Then, the membranes and an appropriate secondary antibody (1:10,000) were incubated at room temperature for 1 h.

### Features of PexD

A Zetasizer instrument was used to test the polydispersity index (PDI), size, size distribution, and zeta potential of PexD in triplicate. Transmission electron microscope (TEM) was used to observe the morphology of Pex and PexD.

### In vitro drug release

PexD was added to thrombin, aPD-L1 was added to a fibrinogen solution, and the solutions were mixed and sprayed through a double-tube spray bottle to form a gel. The formulation was released in PBS medium at pH 6.5 and 37 °C. At the selected time point, the absorbance of DOX was evaluated.

### Cell culture

B16-F10 murine melanoma cells were purchased from the Chinese Academy of Sciences (Beijing, China). The B16-F10 murine melanoma cell line was cultured in high-glucose DMEM supplemented with 10% FBS, penicillin (100 units mL^−1^) and streptomycin sulfate (100 μg mL^−1^). All cells were grown in a 37 °C, 5% CO_2_ cell incubator.

### Adhesion of Pex to cancer cells

A confocal laser scanning microscope was used to observe Pex adhesion to B16-F10 cells. In short, 10^4^ B16-F10 cells were seeded in a 30-mm confocal dish and placed at 37 °C for 24 h. The medium was then replaced with fresh medium, and the confocal petri dish was moved to a 4 °C environment. DiR-labelled Pex were added to the petri dish at 4 °C for 1 h. Then, cold PBS was used to wash the cells 3 times to eliminate unadhered Pex, and pre-incubated in blocking solution (5% non-fat milk in PBS) for 15 min. After being washed with PBS, the cells were incubated with diluted anti-CD44 mAb (1:500) in PBS for 60 min at room temperature. After washing with PBS, the cells were incubated with diluted FITC-conjugated secondary antibody for 60 min at room temperature. Next, 4% formaldehyde was added and incubated with the cells at 37 °C for 10 min. The nuclei were counterstained with Hoechst 33342, and the cells were examined at room temperature.

### Cellular uptake

B16-F10 cells were cultured for 24 h in a 24-well plate at 5 × 10^4^ cells per well. The cells were then incubated for 0.5 or 2 h at 37 °C in fresh medium containing DOX or PexD (DOX concentration of 5 mg mL^−1^). The cells were then continuously incubated with 4% formaldehyde at 37 °C for 10 min before being incubated at room temperature for 10 min with Hoechst 33342. Between each step, the cells were washed three times. Finally, a confocal laser scanning microscope was used to observe the results. For quantitative analysis, the processing was the same as that described above. Flow cytometric analysis was performed on a FACS Calibur instrument to measure cell uptake after cell collection (Becton Dickinson).

### Cytotoxicity assays

The MTT assay was used to assess the antiproliferative activity of DOX and PexD against B16-F10 cells. In 96-well plates, cells (1800 cells/well) were seeded and incubated overnight. In 200 µL fresh culture medium, the cells were exposed to increasing concentrations of drugs for 24 h. Then, an MTT solution was added to the plate and incubated at 37 °C for 4 h. After removing the MTT solution, 100 µL dimethyl sulfoxide (DMSO) was added, and the absorbance at 570 nm was measured using a microplate spectrophotometer.

### HMGB1 and ATP release from cell lines

To investigate the effects of PexD on calreticulin expression in vitro, 2 × 10^5^ B16-F10 cells were seeded in a 35-mm cell culture dish with DOX or PexD at the indicated concentration. The cell culture medium was collected after 24 h of incubation to analyse the released HMGB1 and ATP. To measure HMGB1 and ATP levels in the cell culture medium, western blotting and commercial ATP detection kits (Beyotime Biotechnology) were used.

### ICD-induced maturation of dendritic cells

Bone marrow-derived immature DCs isolated from B16F10 tumour-bearing C57BL/6 mice were co-cultured with B16F10 cancer cells that were pretreated with PexD or DOX. Flow cytometric analysis was performed on a FACS Calibur instrument to measure the maturation status of DCs after cell collection (Becton Dickinson).

### Animal experiments

Mice were housed in the Animal Center of Shenyang Pharmaceutical University (China). All procedures and experiments were carried out in accordance with the guidelines established by our university’s Institutional Animal Ethics Committee (IAEC).

### In vivo elimination of CTCs

CTCs are the main cause of tumour metastasis. To test the ability of PexD to eliminate CTCs, we anaesthetized mice with isoflurane and then injected saline, DOX, platelet-DOX, or PexD (containing 40 μg DOX) via the tail vein. After 30 min, the mice were injected via the tail vein with 1 × 10^6^ B16-F10 cells in 100 µL saline to mimic CTCs. After 14 days, lung tissue was resected for staining and imaging.

### In vivo biodistribution

As described above, B16-F10 tumours were transplanted. Ten days later, about 30% of the tumour were removed to mimic surgery operation and observe biodistribution in tumour readily. The mice were intravenously injected with DiR-labelled Pex or free DiR at a dose of 1 mg kg^−1^ equivalent to DiR (n = 3 for each group) after surgery immediately. At specific time-points, in vivo NIRF images were analyzed with a noninvasive optical imaging system (IVIS) spectrum small-animal imaging system at an excitation wavelength of 748 nm. After 3 h and 12 h post injection, the mice were euthanized to harvest the residual tumours and major organs for analysis with the same IVIS system and settings.

### Studies on post-operative B16-F10 tumour-bearing mouse models

B16-F10 or fLuc-B16-F10 cells (5 × 10^6^) were injected into the right abdomen of female C57BL/6 mice to test the therapeutic effect of aPD-L1-PexD-Gel. This day was regarded as day 0. Ten days later, the tumour reached approximately 500 mm^3^, and the tumour-bearing mice were randomly separated into six groups. The tumour was then partially removed, leaving only approximately 1% of it to represent the small tumours that remain after surgery. In short, isoflurane was used to anaesthetize the mice in an induction chamber, and anaesthesia was maintained via a nose cone. Ninety-nine percent of the tumour was removed using sterile instruments. Immediately following surgery, various formulations of fibrin gel including gel, DOX-Gel, aPD-L1-Gel, PexD-Gel, and aPD-L1-PexD-Gel were sprayed on the surgical tumour bed, and then the surgical site was closed with an autoclip wound clip system. After gel spraying, body weight and tumour volume were evaluated every other day. On day 22, the mice with B16-F10 tumours were sacrificed, the recurrent tumours and major organs were harvested and photographed, and 4% formaldehyde was used to fix tissue for H&E staining. Other mice were fed normally for 48 days, and then each group’s survival rate was assessed. An in vivo bioluminescence imaging system was also used to observe mice inoculated with fLuc-B16-F10 cells. D-luciferin in DPBS (Thermo Scientific Pierce) (15 mg mL^−1^) was injected into the intraperitoneal cavity at a dose of 10 mg mL^−1^ per mouse, and then the IVIS Spectrum Imaging System (Perkin Elmer) was used to image the mice 10 min later. Living Image software was used to quantify the average irradiance (photon s^−1^ cm^−2^ sr^−1^) of the area of interest. When an animal had a poor health status or a tumour size that exceeded 2.5 cm^3^, the animal was euthanized. For the distant tumour model, 5 × 10^6^ B16-F10 or fLuc-B16-F10 cells were transplanted into the right abdomen of female C57BL/6 mice. Tumour cells (1 × 10^6^ B16-F10 or fLuc-B16-F10) were inoculated into the left flank of each mouse. The tumour in the right flank was partially removed 3 days later, and the immunotherapy gel was sprayed on the surgical tumour bed. Next, the tumour was removed as described above.

### Flow cytometry

Tumours were collected from mice and cut into small pieces with surgical scissors before being homogenized in cold staining buffer containing digestive enzymes to form a single-cell suspension. Mouse eyeballs were removed, and blood was collected. Cells were stained with fluorophore-labelled antibodies against F4/80 (BioLegend, Catalogue number 123116), CD206 (BioLegend, Catalogue number 141716, clone C068C2), CD80 (BioLegend, Catalogue number 104722, clone 16-10A1), CD3 (BioLegend, Catalogue number 100204, clone 17A2), CD4 (BioLegend, Catalogue number 100432, clone GK1.5), Foxp3 (BioLegend, Catalogue number 126404, clone MF-14), and CD8 (BioLegend, Catalogue number 100712, clone 53–67) following the manufacturer’s instructions. All of the antibodies were diluted 1:100. The stained cells were run on a flow cytometer (BD FACSCalibur) and analysed with FlowJo software (version 10.4.0). The percentages in flow cytometry plots were used to calculate cell numbers.

### Statistical analysis

The data were performed as mean value ± standard deviation. Statistical comparisons between groups were analyzed using Student’s t-test (two-tailed). Statistical significance was considered at *P < 0.05, **P < 0.01, ***P < 0.001 and ****P < 0.0001.

## Supplementary Information

**Additional file 1: Figure S1. **Oscillatory rheology of aPD-L1-PexD-Gel in (a) dynamic time sweep, (b) dynamic strain sweep, and (c) dynamic frequency sweep. **Figure S2. **Use gel to stick the two kidneys of the mouse together. **Figure S3. **Invitro cytotoxicity after 24 h of different treatments of B16-F10 cells with different concentrations of DOX and PexD. Data are expressed as mean ± SD (n = 3). **Figure S4. **Representative western blot analysis of HMGB1 release from B16-F10 cells treated with PexD or DOX for 24 h at 37 ℃. **Figure S5.** Representative flow cytometric analysis of CD80^+^ CD86^+^ cells. Data are expressed as mean ± SD (n = 3). **Figure S6. **Photos of Bouin’s solution stained whole lungs and H&E staining of the lung slices collected from Tumour, DOX, Platelet-DOX, PexD, Saline groups. Red arrows demonstrate the visible metastatic site. Scale bars: 1 mm. **Figure S7.** In vivo targeting ability of Pex. (A) In vivo biodistribution at different times after i.v. injection of DiR-labeled Pex or an equivalent dose of free DiR. (1 mg kg^−1^) (n = 3) (B) Fluorescent imaging of residual tumours and main organs at 3 h and 12 h post-injection, respectively. Data are expressed as mean ± SD (n = 3). (H: heart; Li: liver; S: spleen; Lu: lung; K: kidney; T: tumour) (C) Quantitative analysis of fluorescent intensities of residual tumours and major organs at 3 h and 12 h post-injection. Data are presented as mean ± SD (n = 3). ***p < 0.001, ****p < 0.0001 versus control. **Figure S8. **Photos of Bouin’s Fluid staining for lungs and H&E staining for major organs slices. The yellow and red arrows demonstrate the lung and liver metastases. Scale bars: 1 mm. **Figure S9. **(A, B) Tumours and spleens after different treatments (n = 5). (C, D) Quantitative graph of tumours and spleens weight. Data are expressed as mean ± SD (n = 5). (C, D) ****p < 0.001 Scale bars: 1 cm.

## Data Availability

All data generated or analyzed during this study are included in this published article.

## Abbreviations

Pex: Platelet derived small EVs
PexD: Small EV-loaded doxorubicin
DOX: Doxorubicin
aPD-L1: Anti-PD-L1 monoclonal antibody
ICIs: Immune checkpoint inhibitors
CTCs: Circulating tumour cells
DiR: 1,1′-Dioctadecyl-3,3,3′,3′-tetramethylindotricarbocyanine iodide
PBS: Phosphate buffered solution
PDI: Polydispersity index
TEM: Transmission electron microscope
ICD: Immunogenic cell death
DCs: Dendritic cells
MTT: 3-(4,5-Dimethylthiazol-2-yl)-2,5-diphenyl tetrazolium bromide
DMEM: Dulbecco’s modified eagle medium
FBS: Fetal bovine serum
DMSO: Dimethyl sulfoxide
H&E: Hematoxylin and eosin
