# Correction: Inhibition of post-surgery tumour recurrence via a sprayable chemo-immunotherapy gel releasing PD-L1 antibody and platelet-derived small EVs

**DOI:** 10.1186/s12951-025-03867-0

**Published:** 2025-12-20

**Authors:** Jian Zhao, Hao Ye, Qi Lu, Kaiyuan Wang, Xiaofeng Chen, Jiaxuan Song, Helin Wang, Yutong Lu, Maosheng Cheng, Zhonggui He, Yinglei Zhai, Haotian Zhang, Jin Sun

**Affiliations:** 1 College of Pharmacy, Shenyang Pharmaceutical University, 103 Wenhua Road, Shenyang, 110016 Liaoning People’s Republic of China; 2 Department of Pharmaceutics, Wuya College of Innovation Shenyang Pharmaceutical University, 103 Wenhua Road, Shenyang, 110016 Liaoning People’s Republic of China; 3 Institute of Robotics & Intel ligent Systems (IRIS) Multi-Scale Robotics Lab (MSRL), ETH Zurich, Zurich, 8092 Switzerland; 4 Key Labora tory of Structure-Based Drug Design & Discovery of Ministry of Education, Shenyang Pharmaceutical University, Shenyang, 110016 China; 5 Department of Biomedical Engineering School of Medical Devices, Shenyang Pharmaceu tical University, Shenyang, 110016 Liaoning China; 6 School of Life Science and Biopharmaceutics, Shenyang Pharmaceutical University, 103 Wenhua Road, Shenyang, 110016 Liaoning People’s Republic of China

**Correction: Journal of Nanobiotechnology (2020) 20:62**

10.1186/s12951-022-01270-7

In this article, Fig. 5A appeared incorrectly. An incorrect version of the image was uploaded during submission. For completeness and transparency, the incorrect and correct versions of Fig. 5 are displayed below. 

Incorrect fig. 5



**Fig. 5** The gel could prevent B16-F10 tumour recurrence after surgery. **A** In vivo bioluminescence imaging of B16-F10 tumour after primary tumour removal. Each treatment group showed four representative mice. The images related to day 10 were taken before the operation. **B**, **C** Average tumour growth kinetics in different groups. When the first mouse in the respective group died, the growth curve stopped. The data are expressed as mean ± SD (*n* = 6). Statistical significance is obtained through multiple comparisons between one-variance analysis and Tukey’s post-hoc test. **D** After the various treatments, the survival rate corresponded to the tumour size of the mice. The data are expressed as mean ± SD (*n* = 6). **E** Changes in body weight of mice in different groups. The data are expressed as mean ± SD (*n* = 4). **F**, **H** Tumours and spleens after different treatments (*n* = 5). Scale bars: 1 cm. **G**, **I** Quantitative graph of tumour and spleen weight. The data are expressed as mean ± SD (*n* = 5). **G**, **I** *P < 0.05; **P < 0.01; ****P < 0.0001

Correct fig. 5

**Fig. 5:**
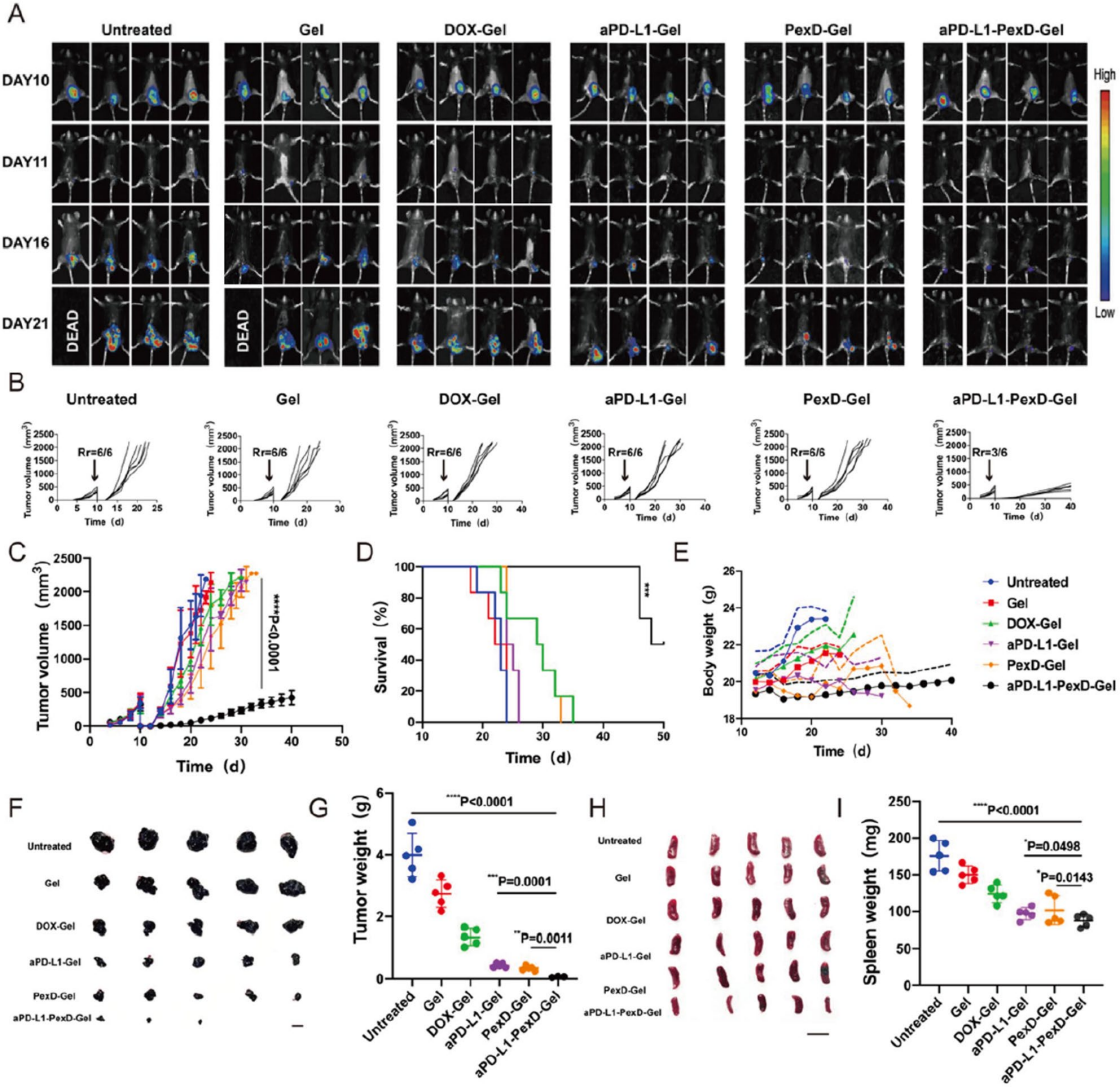
The gel could prevent B16-F10 tumour recurrence after surgery. **A** In vivo bioluminescence imaging of B16-F10 tumour after primary tumour removal. Each treatment group showed four representative mice. The images related to day 10 were taken before the operation. **B**, **C** Average tumour growth kinetics in different groups. When the first mouse in the respective group died, the growth curve stopped. The data are expressed as mean ± SD (*n* = 6). Statistical significance is obtained through multiple comparisons between one-variance analysis and Tukey’s post-hoc test. **D** After the various treatments, the survival rate corresponded to the tumour size of the mice. The data are expressed as mean ± SD (*n* = 6). **E** Changes in body weight of mice in different groups. The data are expressed as mean ± SD (*n* = 4). **F**, **H** Tumours and spleens after different treatments (*n* = 5). Scale bars: 1 cm. **G**, **I** Quantitative graph of tumour and spleen weight. The data are expressed as mean ± SD (*n* = 5). **G**, **I** *P < 0.05; **P < 0.01; ****P < 0.0001

The original article has been corrected.

