# Supplementary material for: Inhibition of post-surgery tumour recurrence via a sprayable chemo-immunotherapy gel releasing PD-L1 antibody and platelet-derived small EVs
Source: J Nanobiotechnology. 2022 Feb 2;20:62. doi: 10.1186/s12951-022-01270-7 (PMC8812025; doi:10.1186/s12951-022-01270-7)
Supplement: Supplementary file 1 — Additional file 1: Figure S1. Oscillatory rheology of aPD-L1-PexD-Gel in (a) dynamic time sweep, (b) dynamic strain sweep, and (c) dynamic frequency sweep. Figure S2. Use gel to stick the two kidneys of the mouse together. Figure S3. Invitro cytotoxicity after 24 h of different treatments of B16-F10 cells with different concentrations of DOX and PexD. Data are expressed as mean ± SD (n = 3). Figure S4. Representative western blot analysis of HMGB1 release from B16-F10 cells treated with PexD or DOX for 24 h at 37 ℃. Figure S5. Representative flow cytometric analysis of CD80+ CD86+ cells. Data are expressed as mean ± SD (n = 3). Figure S6. Photos of Bouin’s solution stained whole lungs and H&E staining of the lung slices collected from Tumour, DOX, Platelet-DOX, PexD, Saline groups. Red arrows demonstrate the visible metastatic site. Scale bars: 1 mm. Figure S7. In vivo targeting ability of Pex. (A) In vivo biodistribution at different times after i.v. injection of DiR-labeled Pex or an equivalent dose of free DiR. (1 mg kg−1) (n = 3) (B) Fluorescent imaging of residual tumours and main organs at 3 h and 12 h post-injection, respectively. Data are expressed as mean ± SD (n = 3). (H: heart; Li: liver; S: spleen; Lu: lung; K: kidney; T: tumour) (C) Quantitative analysis of fluorescent intensities of residual tumours and major organs at 3 h and 12 h post-injection. Data are presented as mean ± SD (n = 3). ***p < 0.001, ****p < 0.0001 versus control. Figure S8. Photos of Bouin’s Fluid staining for lungs and H&E staining for major organs slices. The yellow and red arrows demonstrate the lung and liver metastases. Scale bars: 1 mm. Figure S9. (A, B) Tumours and spleens after different treatments (n = 5). (C, D) Quantitative graph of tumours and spleens weight. Data are expressed as mean ± SD (n = 5). (C, D) ****p < 0.001 Scale bars: 1 cm. [file 12951_2022_1270_MOESM1_ESM.docx]

***Additional file 1***

**Title**

**Inhibition of post-surgery tumor recurrence via a sprayable chemo-immunotherapy gel releasing PD-L1 antibody and platelet-derived small EVs
Authors**:

Jian Zhao1#, Hao Ye23#, Qi Lu2, Kaiyuan Wang2, Xiaofeng Chen2, Jiaxuan Song2, Helin Wang2, Yutong Lu2, Maosheng Cheng4, Zhonggui He2, Yinglei Zhai5, Haotian Zhang6, Jin Sun2*

**Affiliations:**

*1*College of Pharmacy, Shenyang Pharmaceutical University, 103 Wenhua Road, Shenyang Liaoning, 110016, P. R. China
*2*Department of Pharmaceutics, Wuya College of Innovation, Shenyang Pharmaceutical University, 103 Wenhua Road, Shenyang Liaoning, 110016, P. R. China

*3*Multi-Scale Robotics Lab (MSRL), Institute of Robotics & Intelligent Systems (IRIS), ETH Zurich, Zurich 8092, Switzerland.
4Key Laboratory of Structure-Based Drug Design & Discovery of Ministry of Education, Shenyang Pharmaceutical University, Shenyang 110016, China *5*Department of Biomedical Engineering, School of Medical Devices, Shenyang Pharmaceutical University, Shenyang, Liaoning 110016, China

*6*School of Life Science and Biopharmaceutics, Shenyang Pharmaceutical University, 103 Wenhua Road, Shenyang Liaoning, 110016, P. R. China

**#**J.Z. and H.Y. contributed equally to this work.

***Corresponding authors:**Jin Sun, Ph.D.
Tel: Fax: +86-24-23986321

E-mail: sunjin@syphu.edu.cn


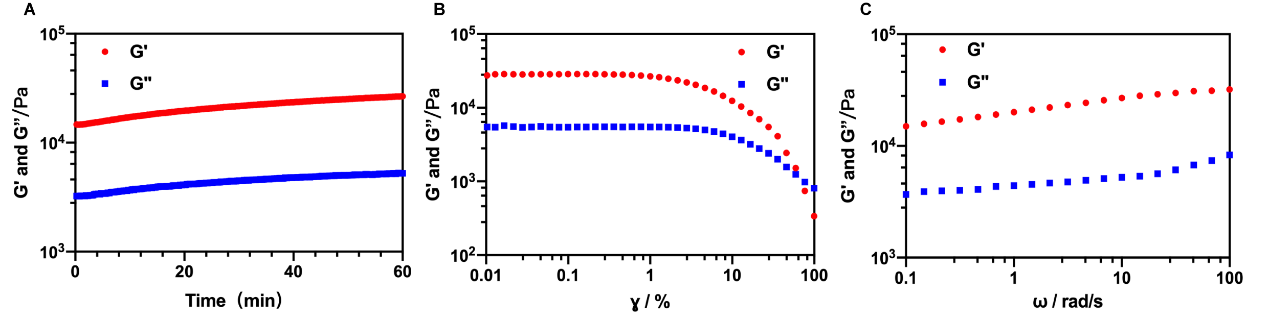


**Fig S1.** Oscillatory rheology of aPD-L1-PexD-Gel in (a) dynamic time sweep, (b) dynamic strain sweep, and (c) dynamic frequency sweep.


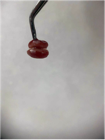


**Fig S2.** Use gel to stick the two kidneys of the mouse together.


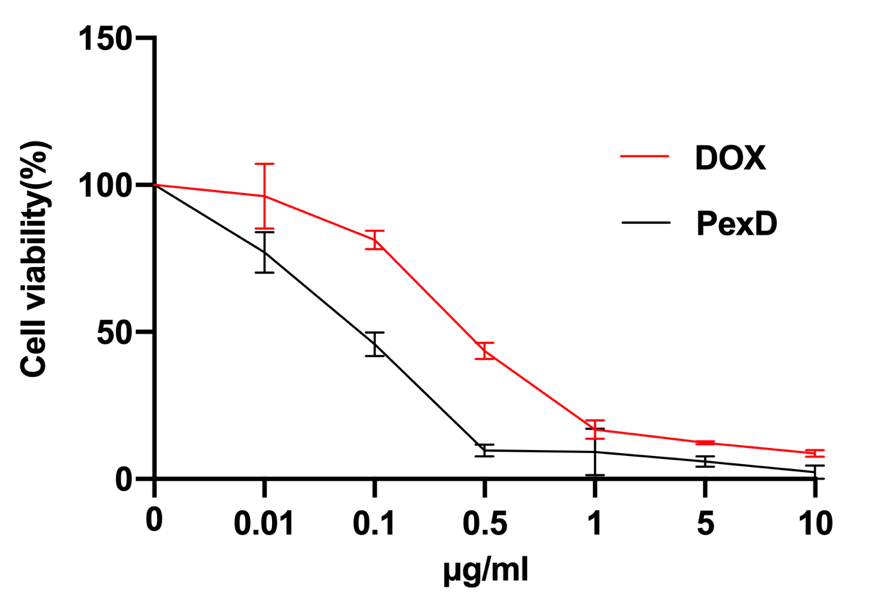


**Fig S3.** Invitro cytotoxicity after 24 hours of different treatments of B16-F10 cells with different concentrations of DOX and PexD. Data are expressed as mean ± SD (n = 3).


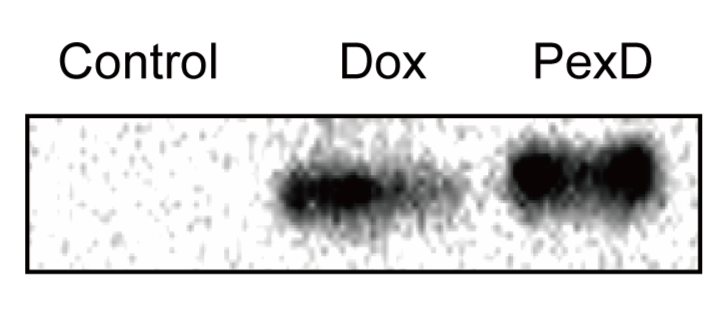


**Fig S4.** Representative western blot analysis of HMGB1 release from B16-F10 cells treated with PexD or DOX for 24 h at 37 ℃.


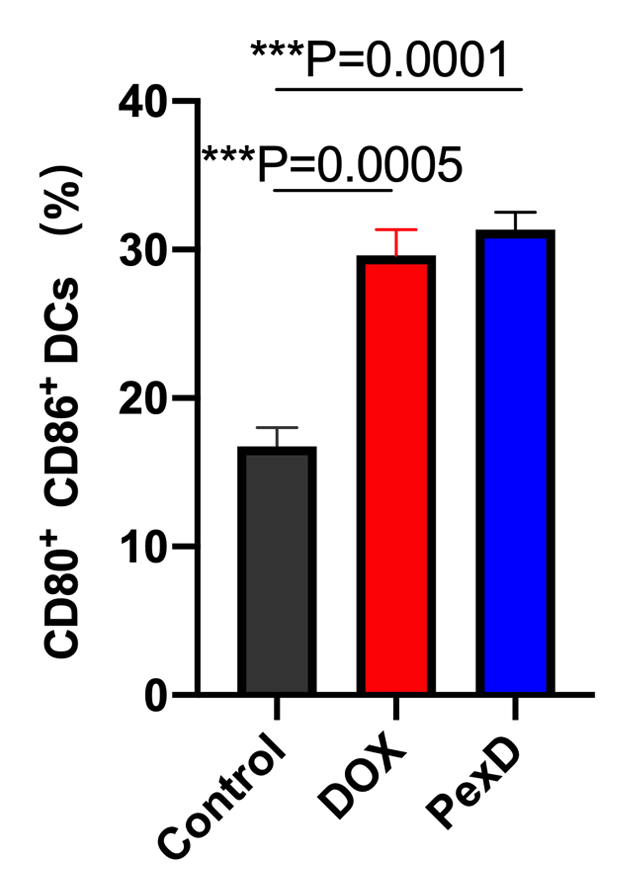


**Fig S5:** Representative flow cytometric analysis of CD80^+^ CD86^+^ cells. Data are expressed as mean ± SD (n = 3).


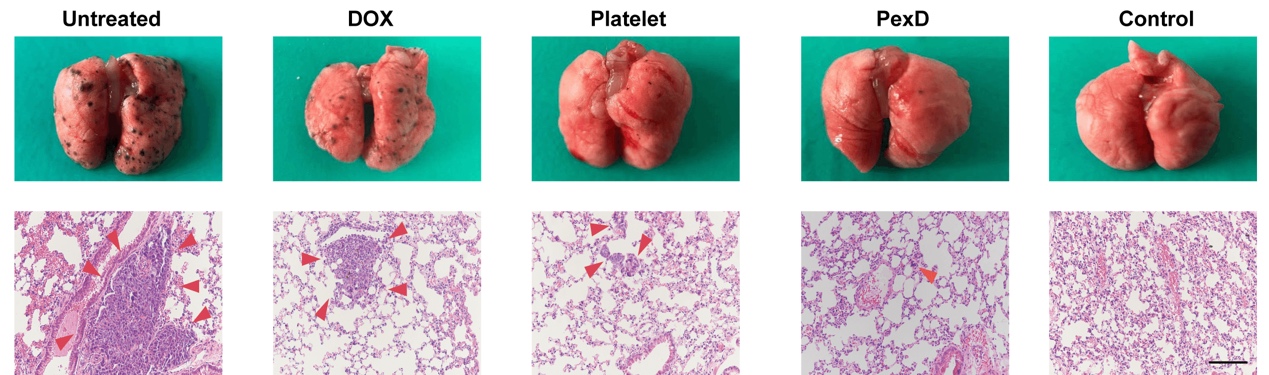


**Fig S6.** Photos of Bouin’s solution stained whole lungs and H&E staining of the lung slices collected fromTumour、DOX、Platelet-DOX、PexD、Saline groups. Red arrows demonstrate the visible metastatic site. Scale bars:1 mm.


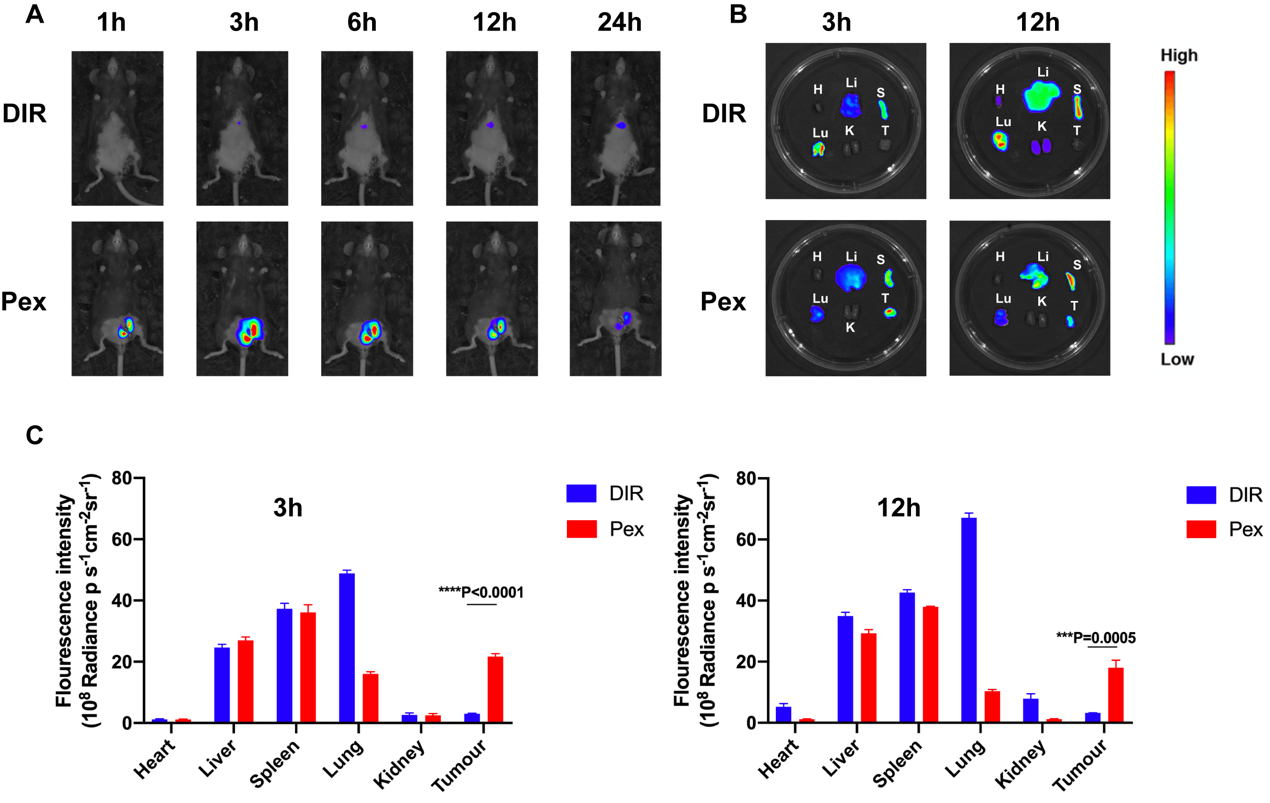


**Fig S7.** *In vivo* targeting ability of Pex. (A) *In vivo* biodistribution at different times after i.v. injection of DiR-labeled Pex or an equivalent dose of free DiR. (1mg kg^-1^) (n = 3) (B) Fluorescent imaging of residual tumours and main organs at 3 h and 12 h post-injection, respectively. Data are expressed as mean ± SD (n = 3). (H: heart; Li: liver; S: spleen; Lu: lung; K: kidney; T: tumour) (C) Quantitative analysis of fluorescent intensities of residual tumours and major organs at 3 h and 12 h post-injection. Data are presented as mean ± SD (n = 3). ***p< 0.001, **** p< 0.0001 versus control.


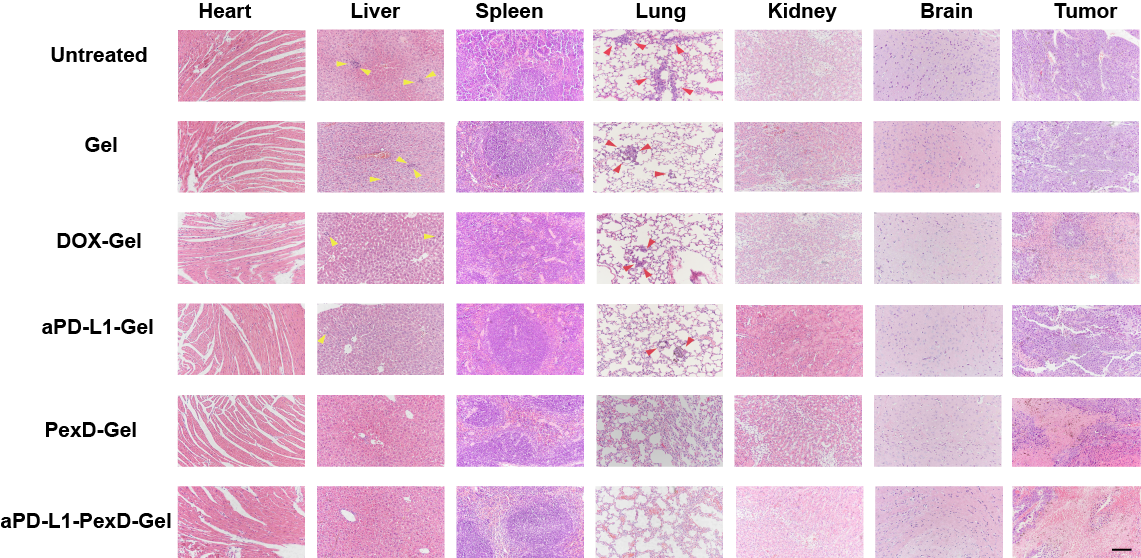


**Fig S8.** Photos of Bouin’s Fluid staining for lungs and H&E staining for major organs slices. The yellow and red arrows demonstrate the lung and liver metastases. Scale bars: 1 mm.


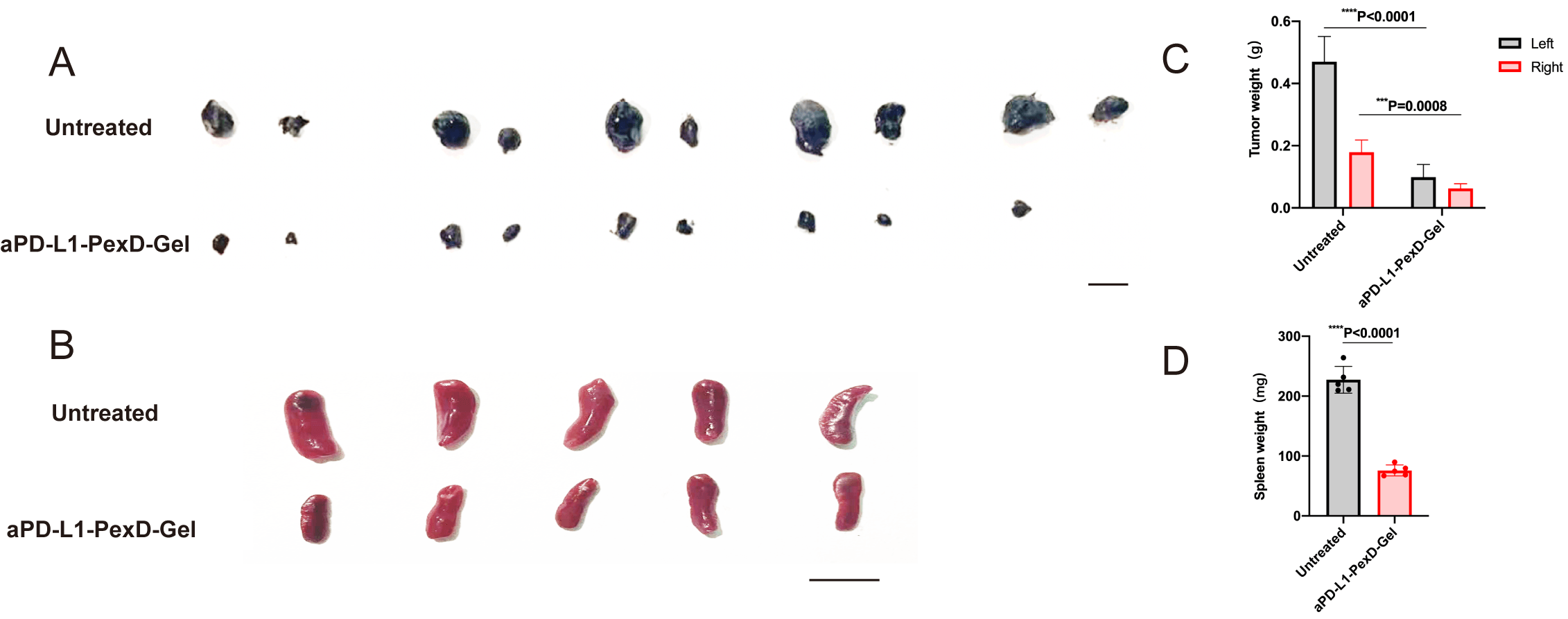


**Fig S9.** (A)(B) Tumours and spleens after different treatments (n = 5). (C)(D) Quantitative graph of tumours and spleens weight. Data are expressed as mean ± SD (n = 5). (C)(D) ****P < 0.001 Scale bars: 1 cm.
